# User satisfaction with the structure and content of the NEXit intervention, a text messaging-based smoking cessation programme

**DOI:** 10.1186/s12889-016-3848-5

**Published:** 2016-11-22

**Authors:** Ulrika Müssener, Marcus Bendtsen, Jim McCambridge, Preben Bendtsen

**Affiliations:** 1 Department of Medical and Health Sciences, Linköping University, Linköping, 581 83 Sweden; 2 Department of Computer and Information Science, Linköping University, Linköping, 581 83 Sweden; 3 Department of Health Sciences, University of York, Heslington, York, YO10 5DD UK; 4 Department of Medical Specialist and Department of Medical and Health Sciences, Motala, Linköping University, Linköping, 581 83 Sweden

**Keywords:** Tobacco, Smoking cessation, Students, Text messages, Mobile phones, SMS

## Abstract

**Background:**

Smoking is still the leading cause of preventable ill health and death. There is a limited amount of evidence for effective smoking cessation interventions among young people. To address this, a text messaging-based smoking cessation programme, the NEXit intervention, was developed. Short-term effectiveness, measured immediately after the 12-week intervention revealed that 26% of smokers in the intervention group had prolonged abstinence compared with 15% in the control group. The present study was performed to explore the users’ experiences of the structure and content of the intervention in order to further develop the intervention.

**Methods:**

Students participating in the main NEXit randomized controlled trial were invited to grade their experiences of the structure and content of the intervention after having completed follow-up. The participants received an e-mail with an electronic link to a short questionnaire. Descriptive analysis of the distribution of the responses to the questionnaire was performed. Free-text comments to 14 questions were analysed.

**Results:**

The response rate for the user feedback questionnaire was 35% (*n* = 289/827) and 428 free-text comments were collected. The first motivational phase of the intervention was appreciated by 55% (158/289) of the participants. Most participants wanted to quit smoking immediately and only 124/289 (43%) agreed to have to decide a quit-date in the future. Most participants 199/289 (69%) found the content of the messages in the core programme to be very good or good, and the variability between content types was appreciated by 78% (224/289). Only 34% (97/289) of the participants thought that all or nearly all messages were valuable, and some mentioned that it was not really the content that mattered, but that the messages served as a reminder about the decision to quit smoking.

**Conclusions:**

The programme was largely perceived satisfactory in most aspects concerning structure and content by young people and most participants stated that they would recommend it to a friend who wants to quit smoking. The motivational phase might be worth shortening and the number of messages around the quit date itself reduced. Shorter messages seemed to be more acceptable.

**Trial registration:**

ISRCTN75766527.

**Electronic supplementary material:**

The online version of this article (doi:10.1186/s12889-016-3848-5) contains supplementary material, which is available to authorized users.

## Background

Although the number of smokers is decreasing in most populations in high income countries around the world, smoking is still the leading cause of preventable ill health and death. The ratio between death and ill health due to smoking is 1:20; thus smoking may lead to premature death, and it is also a major contributing factor of ill health [1]. In Sweden, smoking is responsible for approximately 9.6% of the total disease burden, which means that that around 6000 people die every year due to smoking [2]. The number of young people aged 16–29 years who smoke has been stable for the last 5 years; approximately 11–13% of young women and 7–10% of young men are daily smokers and twice as many are occasional smokers [3].

There is a limited amount of evidence for effective smoking cessation interventions among young people and a prevailing reluctance to seek help in stopping smoking. To address this, a text messaging-based smoking intervention, the NEXit intervention, was developed, aimed specifically at this group [4, 5]. A text messaging-based intervention was chosen because mobile phones are increasingly being used as a medium for reminders about medication and self-management of chronic diseases, such as diabetes, as well as behavioural change interventions on problematic drinking, obesity, tobacco cessation and sedentary lifestyle [6–9]. Young people use mobile phones extensively, therefore this mode of delivery was deemed especially suitable for overcoming barriers to treatment by offering easy access at all times.

The formative development and content of the NEXit 12-week intervention has been reported previously [4], following the steps recommended by Abroms et al. [9]. As well as developing the content based on existing evidence for effective smoking cessation messages, we also analysed the behaviour change techniques (BCTs) included in the intervention using the BCT taxonomy [4, 9], as suggested by Mitchie et al. [10]. The next step was to test the effectiveness of the intervention in a randomized controlled trial (RCT), which included 1590 participants recruited from all colleges and universities in Sweden. The short-term effect, measured immediately after the 12-week intervention, revealed that 25.9% of smokers in the intervention group had prolonged abstinence (not having smoked in the last 8 weeks) compared with 14.6% in the control group [5]. This does not mean that the intervention was flawless and there is no room for improvement from the individual user’s perspective, so the present study was performed to get information for subsequent revisions, thus adding user knowledge to optimize the text-based intervention.

## Methods

The aim of the present study was to explore the users’ experiences of the structure and content of the NEXit smoking cessation intervention. Specifically, we report to what extent the users read the text messages, and explore their views on the content to get user input for further development of the intervention.

### Short description of the NEXit intervention

The NEXit intervention was developed in several steps based on existing recommended smoking cessation manuals used in Sweden [4]. Using the BCT taxonomy suggested by Mitchie et al. [10], which includes 43 BCTs previously found to be reliable in coding the context of smoking cessation interventions, the intervention used 32 different BCTs, for example: “provide information on consequences of smoking and smoking cessation”, “prompt review of goals”, and “boost motivation and self-efficacy”. Slightly more than half of the messages clearly combined more than one BCT [5] (see Table 1). As part of the formative development, 10 smoking cessation experts and 10 smokers evaluated and provided comments and suggestions on each message [4]. The content of the intervention was then revised. Thereafter, a shortened version (8 weeks) was evaluated in a pilot study to test and improve the technical platform developed by the research group.

**Table 1:** Baseline characteristics of the responders and non-responders in the follow-up

Variables	Non-responders (*n =* 538)	Responders (*n =* 289)	Significance
Gender, n (% female)	367 (68.2)	206 (71.3)	NS
Age, n (%)			NS
< 21 years	46 (8.6)	24 (8.3)	
21–25 years	245 (45.5)	127 (8.3)	
26–30 years	118 (21.9)	63 (21.8)	
≥ 31 years	129 (24.0)	75 (26.0)	
Marital status, n (% single)	288 (53.5)	138 (47.8)	NS
Duration of smoking in years, median, (IQR)	8 (7)	8 (8)	NS
Number of cigarettes per week^a^ median (IQR)	70 (70.0)	56 (57.8)	*P =* 0.016^b^
Using snuff, n (% yes)	127 (23.6)	67 (23.2)	NS
Importance of quitting smoking, median (IQR) *(Scale: 1–10 where 1 = not important at all, 10 = very important)*	9 (3)	9 (2)	NS

^a^24 participants did not state how many cigarettes they smoked. Percentages for this variable is based on those that did state the number of cigarettes smoked. Percentages for all other variables are based on the full sample

^b^Mann–Whitney *U* test indicated that the distributions in the two groups differed significantly (Mann–Whitney U = 81292, *P =* 0.016)

The intervention included the following elements: making a public declaration about quitting (i.e. telling friends about the quit attempt), encouraging asking for support from family and friends, distraction techniques, and the possibility of requesting more text messages when the participant experienced strong cravings or a temporary relapse. The intervention began with a motivational phase with 2 SMS (short message services) per day. The messages in the motivational phase contained relevant information in advance of quitting i.e. symptoms to expect on quitting, tips to avoid weight gain, tips to cope with cravings and to avoid smoking triggers, motivational support, and how to distract one’s mind from smoking. The motivational phase lasted for a minimum of 1 week and a maximum of 4 weeks pending on the participant’s preference. During this motivational phase, participants were free to decide when to set a quit date [4, 5]. If no quit date had been set after 4 weeks, participants were given a quit date 3 days later. The core programme started on the quit date and, lasted for 12 weeks.

The 12-week core programme consisted of 157 messages. At participants’ discretion, extra messages were sent regarding cravings, temporary relapses or concerns about weight gain. During the last 3 days of the motivation phase, participants were sent 5 messages per day in order to prepare the participants for the quit day with tips on getting rid of cigarettes and ashtrays, writing a list of reasons for quitting and renewed information on how the body might react to abstinence. The core programme started with 4 messages per day. In weeks 2–4, the participants received 2 messages per day, which was reduced to 2 messages every second day during weeks 5–7. The number of messages per day was reduced to 1 in the final weeks [4, 5].

The messages were sent from a GSM modem and administered from a web-based technical platform that has been developed by one of the authors (MB) and jointly owned by 2 of the authors (PB and MB). Using this platform, sending of all SMS was fully automated, including the extra SMS requested by the participants.

### Study population and procedure

For this study, college and university students participating in the main NEXit RCT were invited to give feedback on the intervention after completing the 12-week intervention, and after participating in the formal follow-up of the RCT [4, 5]. The participants were recruited from all colleges and universities in Sweden except 1 university that participated in a pilot study. The participants came from all levels and disciplines [4, 5]. All participants gave informed consent to participate by clicking on a link in an e-mail invitation and the study was approved by Regional Ethical Committee in Linköping, Sweden (Dnr 2014/217-31). A total of 827 participants were allocated to the intervention group and 763 to the control group (a waiting list group that got access to the intervention after the follow-up). In the main study, we managed to get information on the primary outcome from approximately 95% of the participants in the intervention group and 94% of the controls [5]. This was achieved by sending a high number of reminders over a 4-week period. Non-responders to the follow-up were sent up to 6 reminders by e-mail and 3 reminders by SMS, and 10 attempts were made to phone those who had still not responded.

After this intensive follow-up procedure, we then offered the intervention group the opportunity to give their opinion on the structure and content of the intervention. The intervention group received an e-mail with an electronic link to a short questionnaire with 2 reminders sent 1 week apart to non-responders.

### Questionnaire

The participants from the NEXit study intervention group were asked 14 questions, with 5–7 fixed response options (Additional file 1). The first 2 questions were about change in smoking habits and possible reasons for having stopped smoking or smoking less if applicable. A free-text option gave the participants an opportunity to describe other factors of importance not covered by the fixed response options *(response options: participation in the study/people around me urged me to stop/negative consequences of smoking/too expensive to smoke/ don´t know why I managed to quit).*

Experiences of the structure of the intervention was explored by 6 questions: (1) Perception of the usefulness of the 1–4 week motivation phase (response options: large support/some support/weak support/no support/don´t know 2) Having to set a stop date within the next 4–6 days *(Response options: very simple/simple/somewhat difficult/difficult /don´t know)* (3) perceptions of the large numbers of messages 3 days before the quit date *(response options: far too many/somewhat too many/just right/somewhat too few/too few/don´t know).* (4) the mix of motivating, supporting and factual messages *(response options: very good/good/not particular god/bad/don´t know*). (5) the helpfulness of the option to receive extra messages *(response options: positive and something I used/positive but I chose not to use it/negative, I used the function but did not benefit from it/negative, but I did not use it/don´t know).* (6) how the participants experienced the total duration of the intervention *(response options: far too long /somewhat too long/just right/somewhat too short/too short/don´t know).*

Experiences of the content of the intervention was explored by 5 questions: (1) the perceived usefulness of the content of the messages in the 4-week motivation phase *(response options: very good/good/not particular god/bad/don´t know*); (2) the perceived usefulness of the content of the 12 week core-program *(response options: very good/good/not particular good/bad/don´t know*); (3) the proportion of the total numbers of messages that the participants perceived to be useful *(response options: all/nearly all/about half/some/nearly none/none/don´t know).* Two questions were asked as a proxy for satisfaction with the content; (4) what proportion of all messages were read (response options: *all/nearly all/about half/some/nearly none/none/don´t know); (*5) would the participants recommend the intervention to a friend who ought to quit smoking (response options: yes/no, definitely not/don´t know).

The last question explored whether the participants had used any additional support during the intervention *(response options: no, I did not need additional support/yes, I needed and used additional support (type of support was to be specified)).*

### Data analysis

Descriptive analysis of the distribution of the responses to the 14 questions was performed. In a first step of the analysis all free-text comments to each question were read through by the first and last author (UM and PB). In the second analysis step, the free-text comments were discussed between of the authors and the comments that captured the main content of the specific question with regard to the aim of the study were chosen. The free-text comments are used to underline and illustrate the pattern of response to the fixed response options. The figure after each comment represents the code that were assigned to each of the respondents.

## Results

The response rate was 35% (*n* = 289/827). The baseline characteristics of the participants was similar to non-participants concerning sex, age, marital status, duration of smoking in years, proportion using snuff and perceived importance to quit smoking (Table 1). However, the responders smoked significant fewer cigarettes per week (56 cigarettes (IQR 57.8)) compared to non-responders (70 cigarettes (IQR 70.0)).

Just under half (45%) of the participants provided 428 comments to the 14 questions; the other 55% did not offer any additional comments. Most comments were on the question about what kind of other help the participants had sought during the intervention (*n* = 87). The lowest number of comments were provided for the questions about the number of messages close to the quit date (*n* = 16). On average, around 30 comments were received for each question.

Our findings are presented under the headings presented in the Methods section. We report the responses to the relevant questions and include citations from the free-text comments for each heading.

### Changes in smoking habits and reasons for smoking cessation

A total of 73/289 participants (25%) smoked at the same level as previously, 116 (40%) stated that they were smoking less, 84 (29%) stated that they had quit smoking (compared with 26% in the intervention group as a whole), 9 (3%) participants stated that they now smoked more than before and 7 (2%) answered that they did not know. A number of participants expressed gratitude in the free-text comment for help they received in quitting smoking during the study.

When I received the first message from the programme, I said to myself, “let’s do it.” From that day until now, over a year later, I have smoked 3 cigarettes. No nicotine gum since the New Year, and I don’t smoke any longer. (275)

Most of the participants who stopped smoking during the intervention (*n* = 84 of 289) gave negative consequences as the main reason for quitting smoking (54%); 31% attributed participation in the study and receipt of the intervention as their main reason. Among the negative consequences mentioned was being a poor role model for children, frequent colds and coughs, bad breath/smell and environmental pollution. Some also suggested that trying to smoke on fewer days worked as positive reinforcement because they felt better on days when they did not smoke.

Of course, the driving force to quit smoking was mainly the negative consequences of my role as a parent and the health, environment, economy, but it was really thanks to the messages that I succeeded in quitting. (60)

Especially, I have noticed the positive effects of not smoking. On days when I do not smoke everything is really lovely! To participate in the study was an incredible incentive! (84)

I had a constant cold which was motivation enough to stop smoking. And the fact that my condition changed for the worse and I got smoker’s cough. (214)

### Perception of the experiences of the structure of the intervention

The intervention started with a motivational phase of up to 4 weeks before setting a quit date, depending on the individual’s preference. Just over half (158/289, 55%) of the participants agreed that having the motivational phase before the quit date provided good or very good support before setting a quit date. On the other hand, 40% (*n* = 115/289) did not agree; some thought the messages were repetitive and some said they could be unhelpful reminders about cigarettes. Among those who did managed to quit smoking, 84/289 (67%) stated that the motivational phase gave good or very good support.

That I myself could decide was good because I was in control of when I would stop and be able to plan a little in advance. (52)

I experienced it only as sick tedious. It was just the same things that others say to you when they want you to stop smoking, and that has never motivated anyone to quit.(19)

At any point during the motivational phase, participants had the option to decide a quit date within 4–6 days. Just less than half, 124/289 of the participants (43%), found this structure with 3 days preparation before the quit date as constraining and did not see why they could not should stop smoking immediately.

At first, I thought it was tough, and I felt very pressured when I got the first message. But on the other hand, to set a stop date so close in time was good I think. (46)

Because it is not possible to brush it aside so to speak, like I have done other times when I tried to quit smoking. (30)

It was nice to be able to decide, to plan what day I would stop so I did not have any important commitment the first days after the stop date. (52)

I do not think it was specifically this process of setting a stop date that made it difficult, but rather the fact that you actually decide that after this date, I’ll never smoke again. It’s like breaking up with your best friend on that particular date. (164)

I felt a bit frustrated. I wanted to set a stop date the same day. Why wait? (70)

Nearly half, 47% (*n =* 137), thought there were too many messages during the 3 days before the quit date. However, a similar proportion found the number to be about right. Among those, 20 participants suggested better timing of the messages so they would receive them in relation to meals and social situations when smoking might be most likely.

Very good variety. However, I understand that you don’t want to “disturb” too much. But imagine a smoker’s weekday, for example, the need to receive a text message early in the morning, at 12:30 when lunch is served, at the “two forty-five coffee” for the worker, after supper, and the “late noon cigarette”. Maybe the programme should be more adjustable in relation to weekdays and weekends also. If you could you solve that, I think you would really create something unique. (70)

The variation in the content of the messages between facts and motivational and practical advice was appreciated by most (224/289) of the participants (78%) and particularly among those who quit smoking, the great majority agreed that the variation was good or very good. The participants underlined the need for different content and support over time.

I thought it was very good, I never knew what was coming and the variation contributed so that I didn’t become tired of the messages. (28)

Some days you need more motivation, and other days it was very nice and good that you got a message that was supportive! So I definitely think it was great that the purposes of the messages that I received were different. (46)

A total of 122/289 (42%) of the participants requested, at least once, extra messages concerning relapse, craving or fear of gaining weight. Mean numbers of request for extra messages was 2,4 times (range 1–13) during the entire intervention time. The option to get extra messages was reported as a resource, but a technical delay in message delivery was experienced as disappointing.

The duration of the 12 weeks’ intervention was perceived to be adequate for about half of the participants. Thirty-eight participants (13%) stated that it was too short and 69/289 participants (24%) stated that it was too long.

### Perception of the experiences of the content of the intervention

Concerning the content of the motivational messages, 65% (*n* = 158/289) of all participants found the content very helpful/helpful. A number of participants emphasized that the messages increased their motivation and helped them make a decision. Some thought that the messages were helpful initially but then became less useful, and suggested reducing the number of messages. In general, short messages were perceived as most helpful and the longer messages tended to be seen as repetitive. Some participants who were still smoking perceived the messages as impersonal and more formulated to frighten people.

The messages were good with support and advice as well as motivational information such as what happens when one starts and stops smoking. The messages helped me to stand by my decision to quit smoking. (257)

Most of the messages were very long, and very numerous also. It got a little too much and too repetitive. (20)

I thought the short, more supportive messages were very good. (138)

The majority, 199/289 participants (69%), found the content of the 12 week core programme good or very good. Among those who liked the content, some emphasized that the messages changed their thinking about smoking and they were reminded about why they wanted to quit smoking. Among those 25% who did not value the content, some were concerned that the messages were not tailored to the individual person and certain messages were too basic and just common sense.

Good! The messages helped me to remember why am I doing this, and why I feel irritated, and that cravings don’t last forever! (227)

The messages did not seem appealing to me. I would prefer messages that were more suited to me and my habits, personally. (88)

However, not all messages were perceived as helpful. Only 97/289 participants (34%) thought that all/nearly all messages were valuable. Ten participants suggested that it was not really the content that mattered but the fact that the messages served as a reminder of their decision to quit smoking.

Perhaps 70% of the ones I read were useful; but for me it was not the content itself, but the fact that the messages arrived and reminded me that “yeah, I’m not a smoker”. (134)

The majority of participants (*n* = 196/289, 68%) stated that they had read all/nearly all messages. Only 20/289 participants (7%) said that they only read a few of the messages. Some saved the messages and read them the next day. Most of the participants (*n* = 244/289, 84%) indicated that they would recommend the intervention to a friend and 12% (*n* = 34/289) would not.

If a person is really motivated, yes. I was not as motivated, which is why the messages did not give as good results, as I believe they would have if I were more motivated. (190)

Yes! Great programme! Made me quit smoking after 10 years! (51)

## Discussion

The aim of this study was to explore the users experiences of the structure and content of the SMS-based NEXit smoking cessation intervention aimed at young people [4]. The effectiveness of the intervention has been reported previously [5]. The user evaluation is seen as the last step in a formative development process as suggested by Abroms et al. [9] when designing a text message intervention. This evaluation offers additional knowledge about the barriers and facilitating factors with regard to how users perceive and react to the structure and content of the intervention that can serve as a basis for further revisions of the intervention before large-scale implementation. Despite the effectiveness of the programme, some participants were not helped by the intervention, some disengaged from the intervention and others found it counterproductive.

Despite the response rate of 35%, participants in this user evaluation study provided valuable information that can be used in further development of the intervention. The proportion who quit smoking in this study and in the main study was similar to most baseline characteristics with the only significant difference concerning numbers of cigarettes smoked per week where participants smoked somewhat less (56 versus 70 cigarettes per week). Still, the participants had smoked for the same number of years (8 years) as non-participants. Thus, the participants in this study were regarded as broadly representative of the participants in the intervention group in the RCT [5].

The content of the intervention was based on existing evidence-based face-to-face practices including components derived from expert guidance and official smoking cessation manuals recommended in Sweden [4]. Thus, the overall structure and content of the message-based intervention was well received by most of the participants, regardless of whether they quit or not. Unsurprisingly, participants who were still smoking were less satisfied with the intervention. One possible conclusion also noted in previous research may be that text-based messaging smoking cessation interventions are more suitable for smokers who are motivated to use these types of programmes, and those who are not fully motivated or not determined to stop smoking may find other types of support more suitable [11]. In particular, participants who were still smoking disliked the motivational phase, experienced by many as unnecessary and unhelpful. Greater frequency of messages immediately before the quit date was perceived as problematic because many wanted to quit immediately after having decided to stop. These observations reflect the limitations imposed by untailored interventions.

The theoretical perspectives underpinning interventions such as this emphasize the need for preparation before quitting [12, 13]. Alternatively, if a person feels ready to stop immediately, then it may be more appropriate to encourage them to do so, as about half of quit attempts are made in the moment the smoker has decided to quit [14, 15]. If medication or some other kind of cessation support is going to be used, one needs to plan in advance. For smokers who prefer to quit abruptly, rather than planning ahead, smoking cessation interventions may need to place greater emphasis on the dynamic nature of motivation, allowing for an immediate start to a quit attempt rather than postponing it for motivating preparatory messages [15, 16]. We designed the structure of NEXit so that the frequency of messages per day/week decreased over time because previous research has shown this to be more effective than a constant number of messages [17]. However, as seen in previous studies, the number of messages at certain periods, especially around the quit date, was perceived by many to be too high and might act as a trigger to keep on smoking [18]. On the other hand, other studies show the opposite; that few participants perceived the number of messages to be too few [11]. Allowing users to set their own preferences for message frequency and duration may be worth exploring.

The intervention was fully automated and did not require the user to provide any information. The variation in content between facts and motivational and practical advice was appreciated by most participants, and the timing of specific messages was perceived as satisfactory. However, the content was not tailored to the individuals’ perceived need for support other than participants being able to ask for additional supportive messages when experiencing cravings, fear of weight gain and after a relapse. As seen in other studies, this function with extra messages was used by a minority [11, 19, 20] but this possibility may still be perceived as important, perhaps creating some sense of tailoring and personal control of engagement. Other participants pointed out the lack of tailoring of the messages. Tailoring messages would increase the technical complexity of implementing the intervention and it remains to be seen if this would increase the effectiveness and cost effectiveness of a smoking cessation intervention. The evidence on whether tailoring messages is useful is complex. In some studies, tailoring internet-based interventions for smoking cessation does not increase quit rates in the long term, and seems not to mediate the effect of the intervention [21] whereas others show benefits of tailoring [22]. The mechanisms of the effect of this intervention remain to be identified; is it the actual content of the messages or is it the frequent reminders and reinforcement of having committed oneself to stop smoking, as was pointed out by some participants. This is important to study further.

As seen in previous studies, a majority of the participants read all/nearly all messages [9, 18] emphasizing the advantage of automatic delivery of a message-based intervention that does not require the user to engage in the intervention by opening an app or logging on to a web site. The study is not without limitations due to the low response rate and relative short questionnaire used to explore the views of the participants. The strength is that the participants were recruited from all colleges and universities in Sweden and also the amount of free-text comments to all questions. However, the optimal duration of the intervention has still to be established. In our study, the duration of the intervention was experienced as adequate for only about half of the participants. Most previous message-based quit smoking interventions have been between 8 and 12 weeks long although some have been somewhat shorter.

## Conclusions

The NEXit smoking cessation intervention seems to have supported an encouragingly high proportion of smokers to quit smoking but might have missed the opportunity to help others. On the basis of this study, we are inclined to reduce the duration of the motivational phase and offer fewer messages on the days immediately before the quit date. In general, shorter messages were more acceptable and longer messages will be avoided. Most participants found the intervention satisfactory and would recommend it to a friend who is ready to set a quit date.

## Additional file

Additional file 1: NEXit evaluation questionaire. (DOCX 20 kb)

## Abbreviations

BCT: Behaviour Change Technique
RCT: Randomized Clinical Trial
SMS: Short Message Service
