# Supplementary material for: User satisfaction with the structure and content of the NEXit intervention, a text messaging-based smoking cessation programme
Source: BMC Public Health. 2016 Nov 22;16:1179. doi: 10.1186/s12889-016-3848-5 (PMC5118888; doi:10.1186/s12889-016-3848-5)
Supplement: Additional file 1: — NEXit evaluation questionaire. (DOCX 20 kb) [file 12889_2016_3848_MOESM1_ESM.docx]

***Question for evaluating satisfaction with the structure and content of the SMS-based NEXit smoking cessation intervention***

**Question 1. Have you changed your smoking habits during the time you participated in the study?**

a) Yes, I am smoking more

b) No, I still smoke the same amount of cigarettes

c) Yes, I am smoking less

d) Yes, I have quitted smoking

e) Don´t know

*Free-text comment:*

**Question 2. If you smoke less or have quitted smoking what has been the most important reason?**

*You could only choose one alternative so choose the alternative that you think is the most important reason.*

a) I am still smoking the same amount or more

b) Participation in study and receiving the messages was an important reason

c) People in my surrounding has told me to stop smoking

d) Due to I have noticed negative consequences of my smoking

e) That I cannot afford smoking

f) Don´t know/other reasons

*Free-text comment:*

**Question 3. You received a number of messages before you decided to set a stop-date. To what extend did these messages support/help you deciding to set a date for quitting?**

a) The messages gave a large support

b) The messages gave some support

c) The messages gave a weak support

d) The messages gave no support

e) Don´t know

*Free-text comment:*

**Question 4. Which response option describes best how you perceived the content of the messages you received before setting a stop-date.**

a) Very good

b) Good

c) Not particular good

d) Bad

e) Don´t know

*Free-text comment:*

**Question 5. In the beginning of the programme you were given the opportunity to set a stop-date within 4-6 days. You could set a stop-date within the first week or wait for an additional 1-3 weeks.**

*How do you perceive this way of choosing your stop-date?*

a) Very simple

b) Simple

c) Somewhat difficult

d) Difficult

e) Don’t know

*Free-text comment:*

**Question 6. After you had set a stop-date you started to receive a large number of messages 3 days before your quit-date. How did you perceive this amount of messages?**

a) Far too many

b) Somewhat too many

c) Just right

d) Somewhat too few

e) By far too few

f) Don’t know

*Free-text comment:*

**Question 7. What is your overall perception of the content of the messages you received after your stop-date?**

a) Very good

b) Good

c) Not particular good

d) Bad

e) Don´t know

*Free-text comment:*

**Question 8. The content of the messages covered different aspects. Some was motivating, others supporting and some offered facts about smoking. How did you perceive this variation of the content?**

a) Very good

b) Good

c) Not particular good

d) Bad

e) Don´t know

*Free-text comment:*

**Question 9. How great proportion of the messages did you read? Make as good estimation as possible.**

a) All

b) Nearly all

c) About half

d) Some

e) Nearly none

f) Don´t know

**Question 10. If you reflect on the intervention as a whole, how great proportion of the messages was useful for you?**

a) All

b) Nearly all

c) About half

d) Some

e) Nearly none

f) Don´t know

**Question 11. How did you perceive the possibility to retrieve extra messages for support when having cravings, relapse or worry for weight gain?**

a) Positive and something I made use of

b) Positive, but I choose not to use it

c) Negative, I used it but it did not give any support to me

d) Negative, but I did not use it

e) Don´t know

*Free-text comment:*

**Question 12. How did you perceive the total length of the 12-week intervention?**

a) Far too long

b) Somewhat too long

c) Just right

d) Somewhat too short

e) Far too short

f) Don´t know

**Question 13. Did you need/use some additional smoking cessation support besides the intervention?**

a) No, I did not need additional support

b) Yes, I needed/used additional support: Describe the kind of support:_________

c) Don’t know

*Free-text comment*:

**Question 14. Do you think that you would recommend the intervention to friend that needs support in quitting smoking?**

a) Yes

b) Perhaps, but somewhat doubtful

c) No, definitely not

d) Don’t´ know

*Free-text comment*

_________________________________________________________________________________
